# Molecular Identification and Echinocandin Susceptibility of *Candida parapsilosis* Complex Bloodstream Isolates in Italy, 2007–2014

**DOI:** 10.1371/journal.pone.0150218

**Published:** 2016-02-26

**Authors:** Grazia Lovero, Elisa Borghi, Stella Balbino, Daniela Cirasola, Osvalda De Giglio, Federica Perdoni, Giuseppina Caggiano, Giulia Morace, Maria Teresa Montagna

**Affiliations:** 1 Department of Biomedical Science and Human Oncology, Hygiene Section, Università degli Studi of Bari “Aldo Moro”, Bari, Italy; 2 Department of Health Sciences, Università degli Studi di Milano, Milan, Italy; University of Pittsburgh, UNITED STATES

## Abstract

The *Candida parapsilosis* group encompasses three species: *C*. *parapsilosis*, *C*. *orthopsilosis*, and *C*. *metapsilosis*. Here, we describe the incidence and echinocandin susceptibility profile of bloodstream isolates of these three species collected from patients admitted to an Italian university hospital from 2007 to 2014. Molecular identification of cryptic species of the *C*. *parapsilosis* complex was performed using polymerase chain reaction amplification of the gene encoding secondary alcohol dehydrogenase, followed by digestion with the restriction enzyme BanI. Minimum inhibitory concentrations were determined using the broth microdilution method according to European Committee for Antimicrobial Susceptibility Testing (EUCAST EDef 7.2) and Clinical Laboratory Standards Institute (CLSI M27-A3) guidelines, and the results were compared with those obtained using the E-test and Sensititre methods. Of the 163 *C*. *parapsilosis* complex isolates, 136 (83.4%) were identified as *C*. *parapsilosis*, and 27 (16.6%) as *C*. *orthopsilosis*. The species-specific incidences were 2.9/10,000 admissions for *C*. *parapsilosis* and 0.6/10,000 admissions for *C*. *orthopsilosis*. No resistance to echinocandins was detected with any of the methods. The percent essential agreement (EA) between the EUCAST and E-test/Sensititre methods for anidulafungin, caspofungin, and micafungin susceptibility was, respectively, as follows: *C*. *parapsilosis*, 95.6/97.8, 98.5/88.2, and 93.4/96.3; *C*. *orthopsilosis*, 92.6/92.6, 96.3/77.8, and 63.0/66.7. The EA between the CLSI and E-test/Sensititre methods was, respectively, as follows: *C*. *parapsilosis*, 99.3/100, 98.5/89.0, and 96.3/98.5; *C*. *orthopsilosis*, 96.3/92.6, 100/81.5, and 92.6/88.9. Only minor discrepancies, ranging from 16.9% (*C*. *parapsilosis*) to 11.1% (*C*. *orthopsilosis*), were observed between the CLSI and E-test/Sensititre methods. In conclusion, this epidemiologic study shows a typical *C*. *parapsilosis* complex species distribution, no echinocandin resistance, and it reinforces the relevance of using commercially available microbiological methods to assess antifungal susceptibility. These data improve our knowledge of the national distribution of species of the *psilosis* group, as there are very few studies of these species in Italy.

## Introduction

*Candida* spp. are important causative agents of nosocomial fungal infections, and they are associated with significant morbidity, prolonged hospital stays, high mortality, and increased healthcare costs. While *Candida albicans* is the most common cause of such infections, recent epidemiologic studies have reported increasing candidemia due to the *Candida parapsilosis* complex [1–3], which has become the predominant yeast in some pediatric and hematology wards [4, 5].

The *C*. *parapsilosis* complex historically has been categorized into groups I, II, or III. However, molecular fingerprinting and mitochondrial genome architectures have demonstrated that these groups correspond to three different species: *Candida parapsilosis sensu stricto* (formerly group 1, referred to hereafter as *C*. *parapsilosis*), *Candida orthopsilosis* (formerly group 2), and *Candida metapsilosis* (formerly group 3) [6, 7].

Although closely related, the members of the *C*. *parapsilosis* complex differ from each other regarding their virulence and echinocandin susceptibility. *In vitro* infection models have suggested that *C*. *metapsilosis* is the least virulent species [8, 9], and this is in line with its low clinical relevance compared with *C*. *parapsilosis* and *C*. *orthopsilosis* [10, 11]. *In vitro* susceptibility data on anidulafungin (AND), caspofungin (CSP), and micafungin (MCF) indicate that *C*. *parapsilosis* is less susceptible than *C*. *metapsilosis* and *C*. *orthopsilosis* [12, 13].

Until now, there has not been an extensive study in Italy concerning the epidemiological data and the echinocandin susceptibilities of species of the *psilosis* group [11, 14]. Therefore, the aim of this study was: i) to describe the candidemia incidences of *C*. *parapsilosis*, *C*. *metapsilosis*, and *C*. *orthopsilosis* in a large university hospital in Southern Italy; ii) to evaluate the susceptibilities of these three species to AND, CSP, and MCF using both the European Committee for Antimicrobial Susceptibility Testing (EUCAST) and the Clinical Laboratory Standards Institute (CLSI) broth microdilution methods; and iii) to evaluate two commercially available methods (E-test and Sensititre) in terms of their agreement with the CLSI and EUCAST methods.

## Materials and Methods

From January 2007 to December 2014, a prospective, observational, laboratory survey of *Candida* bloodstream infections (BSIs) was conducted in a large university hospital in Southern Italy. A case of candidemia was defined as having at least one positive blood culture yielding *Candida* spp. Only the first episode of candidemia was reported for patients with recurrent or subsequent episodes of infection. Detailed data (sex, age, hospital department, and underlying condition) for each case were analyzed. For the purpose of this study, all patients (n = 163) diagnosed with candidemia due to the *C*. *parapsilosis* complex (34.8% of all *Candida* BSIs) were investigated.

The local institutional review committee (Azienda Ospedaliero–Universitaria Policlinico of Bari, Italy) approved the study, and informed consent was not required because of the observational nature of this study. Registered data were managed in accordance with Italian data protection laws (privacy law).

### Strain Collection

The isolates were phenotypically identified as the *C*. *parapsilosis* complex using the ID32C and VITEK-2 System (BioMérieux, Marcy l’Etoile, France), stored in glycerol at −80°C, and cultured on Sabouraud dextrose agar plates (BioMèrieux) to ensure purity and viability prior to molecular identification and antifungal testing.

### Molecular Identification

Prior to nucleic acid extraction, all strains were grown overnight at 35°C in yeast extract-peptone-dextrose medium. Yeast genomic DNA was extracted using the PrepMan Ultra Sample Preparation Reagent (Applied Biosystems, Carlsbad, CA, USA) according to the manufacturer’s protocol. The extracted DNA was quantified with a spectrophotometer by measuring its absorbance at 260 nm, and stored at −20°C until used.

BanI digestion patterns of the secondary alcohol dehydrogenase (SADH) polymerase chain reaction (PCR) products allow the identification of the three species [6]. Briefly, a 716-bp fragment of the SADH gene was amplified by PCR with the primers S1F (5′-GTTGATGCTGTTGGATTGT-3′) and S1R (5′-CAATGCCAAATCTCCCAA-3′), and then digested with the restriction endonuclease BanI. *C*. *parapsilosis*, *C*. *orthopsilosis*, and *C*. *metapsilosis* isolates were identified by differences in the number of restriction sites contained in their SADH amplicons, one, zero (no restriction site), and three BanI restriction sites, respectively. *C*. *parapsilosis* American Type Culture Collection (ATCC) 22019, *C*. *orthopsilosis* ATCC 96139, and *C*. *metapsilosis* ATCC 96143 were included as quality control strains.

### Antifungal Susceptibility Testing

CLSI document M27-A3 [15] and EUCAST document EDef 7.2 [available on the EUCAST website: http://www.eucast.org] methods were described in a previous report [16]. Standard antifungal powders of AND (Pfizer Pharmaceuticals, Groton, CT, USA), CSP (Merck & Co., Inc., Whitehouse Station, NJ, USA), and MCF (Astellas Pharma, Tokyo, Japan) were provided by the respective manufacturers. The E-test assay (AB BIODISK, BioMérieux) using RPMI-1640 agar plates (Biolife, Milan, Italy), and Sensititre YeastOne technique (SYO-09 panel, Trek Diagnostic Systems, Ltd, East Grinstead, England) were performed as instructed in the commercial guidelines.

All tests were performed in duplicate and in case of discrepancies were repeated once more.

Quality control was performed for each method and for each session of testing, using *Candida krusei* ATCC 6258 and *C*. *parapsilosis* ATCC 22019 according to the CLSI M27-A3 document [15].

### Interpretation and Analysis of Results

Statistical analyses were performed with GraphPad Prism version 5.0 for Windows (San Diego, CA, USA). Comparison between the members of *C*. *parapsilosis* complex was based on Student’s t-test for continuous variables and on the chi-square test or Fisher’s exact test for categorical variables. The level of significance was set at a p value less than 0.05.

Echinocandin susceptibilities were defined according to the species-specific clinical breakpoints (CBPs) proposed by EUCAST version 7.0 [available on the EUCAST website: http://www.eucast.org/clinical_breakpoints] and by CLSI M27-S4 documents [17].

The minimum inhibitory concentrations (MICs) for each echinocandin obtained using the EUCAST and CLSI techniques were compared with those obtained using the E-test and Sensititre systems by assessing the essential agreement (EA) and categorical agreement (CA). MIC discrepancies of no more than ± 2-fold dilutions were used to calculate the EA. The E-test MICs were rounded to the next highest CLSI or EUCAST concentration to simplify the comparison. For antifungal agents whose CBPs have been set, CA was defined as the percentage of isolates classified in the same category by the reference procedures and the E-test and Sensititre systems. Discrepancies were considered “very major errors” when the reference method categorized the organism as resistant, but the E-test or Sensititre methods categorized the organism as susceptible. “Major errors” occurred when the reference method categorized the isolate as susceptible, but the E-test or Sensititre methods categorized it as resistant. “Minor errors” (MiEs) occurred when the reference method categorized an organism as susceptible or resistant and the E-test or Sensititre methods categorized it as intermediate, or the reference method categorized it as intermediate and the E-test or Sensititre methods categorized it as susceptible or resistant.

## Results

According to the BanI restriction patterns of the PCR amplicons, 136 (83.4%) strains were identified as *C*. *parapsilosis*, and 27 (16.6%) were identified as *C*. *orthopsilosis*. No *C*. *metapsilosis* strains were identified. The species-specific average incidences were 2.9/10,000 admissions for *C*. *parapsilosis* (incidence range: 2–4.4) and 0.6/10,000 admissions for *C*. *orthopsilosis* (incidence range: 0.3–0.9), without any trend during the study period for both species.

The patients’ demographic and clinical information is summarized in Table 1. The intensive care unit (ICU) was the most frequent ward (55.2%, 90/163), especially for *C*. *parapsilosis* (82/163 cases vs. 8/27 cases for *C*. *orthopsilosis*; p<0.01). Oncohematological disorders were associated more commonly with *C*. *orthopsilosis* than with *C*. *parapsilosis* (12/27 cases vs. 27/136 cases, respectively; p<0.01).

**Table 1:** Characteristics of 163 patients with *Candida parapsilosis* complex candidemia.

Characteristic	*C*. *parapsilosis* (n = 136)	*C*. *orthopsilosis* (n = 27)	p-value
**Male, n (%)**	98 (72.1)	14 (51.9)	0.039
**Age (years) mean (SD)**	44.5±23.7	46± 26.8	0.762
**Hospital ward, n (%)**			
Hematology	8 (5.9)	1 (3.7)	1.000
Adult ICU	57 (41.9)	7 (25.9)	0.123
Internal medicine	15 (11.0)	2 (7.4)	0.741
Neonatal ICU	25 (18.4)	1 (3.7)	0.081
Pediatric onco-hematology	19 (14.0)	11 (40.7)	0.001
Surgery	12 (8.8)	5 (18.5)	0.164
**Underlying condition, n (%)**			
Autoimmune disorder	2 (1.5)	0 (0.0)	1.000
Cancer	13 (9.6)	3 (11.1)	0.731
Central venous catheter	130 (95.6)	23 (85.2)	0.062
Gastrointestinal diseases	11 (8.1)	2 (7.4)	1.000
HIV infection	4 (2.9)	1 (3.7)	1.000
HSCT	4 (2.9)	0 (0.0)	1.000
Kidney diseases	6 (4.4)	0 (0.0)	0.591
Major surgery	21 (15.4)	5 (18.5)	0.774
Onco-hematological diseases	27 (19.9)	12 (44.4)	0.006
Premature birth	14 (10.3)	1 (3.7)	0.469
Pulmonary diseases	6 (4.4)	0 (0.0)	0.591
Sepsis	16 (11.8)	2 (7.4)	0.740
Trauma	12 (8.8)	1 (3.7)	0.698

HSCT, Hematopoietic Stem Cell Transplant; ICU, Intensive Care Unit.

The antifungal susceptibility testing results, as well as the percent EA for each echinocandin, are summarized in Table 2. When the species-specific CBPs were applied, no resistance was detected with any of the tested methods. The geometric mean MICs (μg/mL) by both methods (EUCAST/CLSI) for AND, CSP, and MCF was, respectively, as follows: *C*. *parapsilosis* 1.79/1.24, 1.84/1.74, and 1.77/1; *C*. *orthopsilosis* 1.85/1.08, 1.90/1.26, and 1.76/0.58.

**Table 2:** MICs and EAs of the E-test and Sensititre methods compared with those of the CLSI and EUCAST methods.

Isolates (No.)	Method	Anidulafungin (μg/ml)				Caspofungin (μg/ml)				Micafungin (μg/ml)			
		MIC range	MIC_50/90_	% EA, with respect to EUCST CLSI		MIC range	MIC_50/90_	% EA, with respect to EUCST CLSI		MIC range	MIC_50/90_	% EA, with respect to EUCST CLSI	
***C*. *parapsilosis* (136)**	EUCAST	0.5–2	2/2			1–2	2/2			0.12–2	2/2		
	CLSI	0.5–2	1/2			0.25–4	2/2			0.008–2	1/2		
	E-test	0.25–4	1/2	95.6	99.3	0.25–4	1/2	98.5	98.5	0.008–2	0.5/1	93.4	96.3
	Sensititre	0.25–2	1/2	97.8	100	0.25–2	0.5/1	88.2	89	0.015–2	1/1	96.3	98.5
***C*. *orthopsilosis* (27)**	EUCAST	1–2	2/2			1–2	2/2			1–2	2/2		
	CLSI	0.5–2	1/2			0.25–2	1/2			0.008–2	0.5/2		
	E-test	0.06–4	1/2	92.6	96.3	0.25–2	0.5/1	96.3	100	0.25–2	0.25/1	63.0	92.6
	Sensititre	0.12–2	0.5/2	92.6	92.6	0.25–1	0.5/1	77.8	81.5	0.25–2	0.5/2	66.7	88.9

CLSI, Clinical and Laboratory Standards Institute; EUCAST, European Committee on Antimicrobial Susceptibility Testing; MIC, minimum inhibitory concentration; EA, essential agreement

*Candida parapsilosis* and *C*. *orthopsilosis* showed similar susceptibility patterns to all echinocandins, with MIC_90_s ranging from 1–2 μg/mL, depending on the method. The MICs of the quality control strains fell within the established ranges that have been published for both methods [15, 18].

For *C*. *parapsilosis*, the EAs between the echinocandin MIC results obtained by the E-test and Sensititre methods and the reference procedures (CLSI and EUCAST) were very high, ranging from 88.2% to 100% according to the technique–drug combination. For *C*. *orthopsilosis*, EAs between the E-test and Sensititre methods and the CLSI method were also high, ranging from 81.5% to 100% according to the technique–drug combination. In comparison to EUCAST, the EA was worse for MCF (E-test, 63.0%; Sensititre, 66.7%) than for AND (92.6% for the E-test and Sensititre methods) and CSP (E-test, 96.3%; Sensititre, 77.8%).

Regarding the CAs between the E-test and Sensititre techniques and the EUCAST method, for AND and MCF, discrepancies were not observed for each organism tested. Comparing the CLSI method with the E-test and Sensititre techniques, for *C*. *parapsilosis* the CAs (%) were 91.9/100, 94.9/96.3, and 100/100 for AND, CSP, and MCF, respectively. Regarding *C*. *orthopsilosis*, the CAs were 100% for CSP and MCF, respectively with exception of AND (88.9%) when the E-test was compared with the CLSI method. Only MiEs occurred, and they were slightly greater for *C*. *parapsilosis* (23/136, 16.9%) than *C*. *orthopsilosis* (3/27, 11.1%). Consequently, 11 *C*. *parapsilosis* and three *C*. *orthopsilosis* isolates classified as AND susceptible by the CLSI method were considered to be intermediate by the E-test. For CSP, ten *C*. *parapsilosis* isolates were categorized as intermediate by the CLSI and susceptible by the E-test and Sensititre (five strains for each reference-commercial method comparison), and two were CLSI susceptible and E-test intermediate.

## Discussion

Candidemia caused by the *C*. *parapsilosis* complex is increasing in Italy [1] and some other countries [2, 3], and it is the second most commonly isolated *Candida* species. Genotypic differences allowed the taxonomic division of the *C*. *parapsilosis* complex into three groups that were recognized as separate species: *C*. *parapsilosis* (group 1), *C*. *orthopsilosis* (group 2), and *C*. *metapsilosis* (group 3). The epidemiology of candidemia and the antifungal susceptibility of these species are scarcely defined in Italy. To our knowledge, only one national study of the epidemiology of invasive candidiasis caused by the *C*. *parapsilosis* complex has been published [11], which showed that 95, 3.6, and 1.4% of the *C*. *parapsilosis* complex strains were identified as *C*. *parapsilosis*, *C*. *orthopsilosis*, and *C*. *metapsilosis*, respectively.

Based on these data, the present study was performed to simultaneously evaluate the epidemiology and echinocandin susceptibility patterns of *C*. *parapsilosis* complex BSIs in a university hospital in Southern Italy from January 2007 to December 2014. At our institution, the incidence of *C*. *parapsilosis* (83.4% of all *C*. *parapsilosis* complex isolates) was 2.9 cases per 10,000 admissions; while incidences of 2.2–2.9 and 3.4 were reported in Spain and Turkey, respectively [10, 12, 19]. The incidence of *C*. *orthopsilosis* candidemia was 0.6 per 10,000 admissions, which is similar to the incidences reported by other studies, which range from 0.2 to 0.9 [10,12]. Interestingly, the percentage of *C*. *orthopsilosis* observed (16.6%) was one of the highest published thus far [11–13, 20, 21], although higher percentages were reported in Spain (23.5%) [10] and Qatar (24%) [22]. There were no cases of *C*. *metapsilosis* candidemia, in keeping with studies performed in Portugal [23], Qatar [22], Scotland [24], and Kuwait [25]. *C*. *metapsilosis* has been reported to be rarely recovered from blood (incidence of 0.7–6.9%) [13, 20], which is in accordance with its low virulence [8, 9]. It is noteworthy that some authors have reported that *C*. *metapsilosis* is more common than *C*. *orthopsilosis* [13, 26]. This suggests a geographical variation in the distribution of species belonging to the *C*. *parapsilosis* complex.

The *C*. *parapsilosis* complex is particularly common in critically ill patients, probably because this yeast has a high affinity for vascular devices, medical instrumentation, and indwelling plastics [27]. In fact, in the present study, the majority of patients (55.2%) infected with the *C*. *parapsilosis* complex were from the ICU, and the indwelling catheter was the most frequent underlying condition (93.9%), and it was mainly observed in the ICU patients (58.8%). Of note, we observed that ICU patients were more likely to develop *C*. *parapsilosis* candidemia than *C*. *orthopsilosis* candidemia. This issue may be partially explained by the greater capacity of this species to form biofilms on central lines [28], compared with the closely related species *C*. *orthopsilosis* and *C*. *metapsilosis* [29].

Concerning the susceptibility results, both CLSI and EUCAST procedures were used in this study, and no echinocandin resistance by the species within the *psilosis* complex was observed using either method, as was reported by other studies [11, 20]. In agreement with results previously reported by Garcia-Effron *et al*. [10], our MICs showed a rank order of activity with MCF> AND> CSP against *C*. *parapsilosis* and *C*. *ortopsilosis*. However, some studies [13, 14] showed that these species are more susceptible to CSP than to the other echinocandins. These data reflect the need of wider study regarding the echinocandin susceptibility. Of note, some authors have reported that *C*. *parapsilosis* is the only species that is resistant to echinocandins [11, 23, 30]. In the current study, the echinocandin MICs for *C*. *parapsilosis* were similar to those for *C*. *orthopsilosis* (MIC_90_ of 2 μg/mL for each organism according to the technique–drug combination), which differ from those reported in a multicenter study conducted in Italy by Borghi *et al*. [11]. This difference may result from the fact that, in the present study, the isolates were only collected in one hospital; thus, the different results could be related to differences in antifungal drug management between the hospitals analyzed in the two studies.

The present study compared, for the first time, the echinocandin MICs obtained by the E-test and Sensititre methods with those obtained by the CLSI and EUCAST procedures by determining the species of the *psilosis* group. Our analysis showed that there were excellent EAs between these methods, except between the EUCAST and the E-test and Sensititre methods when testing the MCF susceptibility of *C*. *orthopsilosis*, which was less than 67%. The meaning of this *in vitro* finding is not clear and needs to be clarified in more detail. A good CA was also observed for all organism-drug combinations, ranging from 88.9 to 100%. The MIC differences between the standard procedures and the commercially available assays are small enough that the choice of method should not result in susceptibilities that differ enough to affect treatment decisions.

As our study was an observational laboratory-based survey, some medical files were missing: the severity of illness scores, the type and duration of antifungal therapy, and mortality data. Nevertheless, to the best of our knowledge, this study provides the first data on the incidence of species of the *psilosis* group that are responsible for candidemia in Italy. Although this study was conducted in a single hospital, we attained a large sample size (more than 45,000 patients). Moreover, this 8-year survey revealed no echinocandin resistance among the species within the *C*. *parapsilosis* complex using the CLSI and EUCAST methods, thereby suggesting that these strains are, in generally, highly susceptible to echinocandins [14, 16]. Finally, a comparison of the CLSI and EUCAST methods and the E-test and Sensititre methods revealed that they yielded similar MICs, which reinforces the relevance of using commercially available methods in clinical microbiology laboratories to test for antifungal susceptibility.
